# Membranous fat necrosis of gall bladder, a case report and literature review

**DOI:** 10.1016/j.ijscr.2023.108796

**Published:** 2023-09-07

**Authors:** Mahsa Salehi, Somayeh Sheidaei, Maryam Nourbakhsh, Shafi Rehman, Mehrnaz Salehi, Hamid Reza Jafari

**Affiliations:** a Mazandaran University of Medical Sciences, Mazandaran, Iran; b Khyber Medical College, Peshawar, Pakistan; c Shahrekord University of Medical Sciences, Shahrekord, Iran; d Isfahan University of Medical Sciences, Isfahan, Iran

**Keywords:** Membranous fat necrosis, Gall bladder, Acute cholecystitis, Histology, Cholangitis

## Abstract

**Introduction and importance:**

Membranous fat necrosis is a rare histological finding. Despite its low incidence and lack of clinical significance, it can involve various organs. Majority of membranous fat necrosis cases are diagnosed in breast lumps and skin in comparison to intra-abdominal lesions. There has been only one reported case of membranous fat necrosis of gall bladder in literature.

**Case presentation:**

A 56-year-old female patient with previous history of diabetes mellitus and hypertension was administered due to abdominal pain and fever. Based on her physical exam, lab data, and ultrasonography, she was diagnosed by cholangitis. After primary care, she went under cholecystectomy. The histological finding of gall bladder revealed crenulated fatty membranes phagocytized by macrophages in Hematoxylin and Eosin (H&E) staining. Moreover, necrosis and giant cells were seen on Sudan black staining. Hence, the diagnosis of membranous fat necrosis in gall bladder was made.

**Clinical discussion:**

Membranous fat necrosis occurs when peripheral blood circulation is compromised. Ischemia of fat tissue cause fatty membranous material accumulation acting as foreign bodies. Hence, it can attract inflammatory response. Regarding pathology, phagocyted membranous by macrophages and giant cells is diagnostic. Sudan black, Luxol fast blue (LFB), long Ziehl-Neelsen, and D-PAS are positive in membranous fat necrosis.

**Conclusion:**

Membranous fat necrosis of gall bladder is a rare entity. This is the second reported case of such diagnosis. Nonetheless, further pathological investigations are necessary.

## Highlights

• Membranous fat necrosis, gall bladder, acute cholangitis

## 1 Introduction

Membranous fat necrosis (MFN), also known as lipomembranous panniculitis and lipomembranous fat necrosis (LFN), is commonly found in skin disorders. However, it is a rare entity in other organs [1]. In 1973, MFN was first introduced in the literature. Consequently, MFN was a histopathological finding in skin disorders such as diabetes mellitus, systemic sclerosis, systemic lupus erythematous, and perineal lipo-granuloma. Scarcely, MFN is found in breast lesions, lipoma, ovarian teratoma, intra-articular loose bodies, cardiac valves, and appendices epiploicae [1,2]. Moreover, there are some reports of MFN findings in pancreatitis patients [[3], [4], [5], [6], [7]]. Obesity, diabetes mellitus, and circulatory disorders are mostly associated with MFN. Regarding its extreme rarity, membranous fat necrosis in gall bladder was only reported in one study [1].

In the following study, we represent the second and most recent case of membranous fat necrosis of gall bladder as well as literature review.

## 2 Case presentation

An obese 56-year-old woman was admitted in the emergency department due to acute abdominal pain. Constant pain was located mostly in the RUQ radiating to all abdominal quadrant. Although she had had previous history of similar pain from 6 months age, this episode of intolerable pain started 3 h before patient's admission after having a large meal. Also, she complained of fever, nausea, and vomiting. Regarding her history, she suffered from insulin dependent diabetes mellitus and hypertension. In her physical exam, she was agitated, dehydrated, feverish, icteric, and tachycardic. She had generalized abdominal tenderness. Lab data showed high total bilirubin and leukocytosis. Ultrasonography showed pericholecystic fluid, wall thickening and edema of 51 mm, and positive Murphy sign. Based on the diagnosis of acute cholangitis, primary pain management, fluid resuscitation, GI rest, and antibiotic therapy were done. After stabilizing the patient and resolving abdominal pain for 3 days, she went under laparoscopic cholecystectomy. She experienced full post-op recovery and she was discharged with no complications. Her follow-up was uneventful. Written informed consent was obtained from the patient's parents for publication and any accompanying images. A copy of the written consent is available for review by the Editor-in-Chief of this journal on request.

**Fig. 1:**
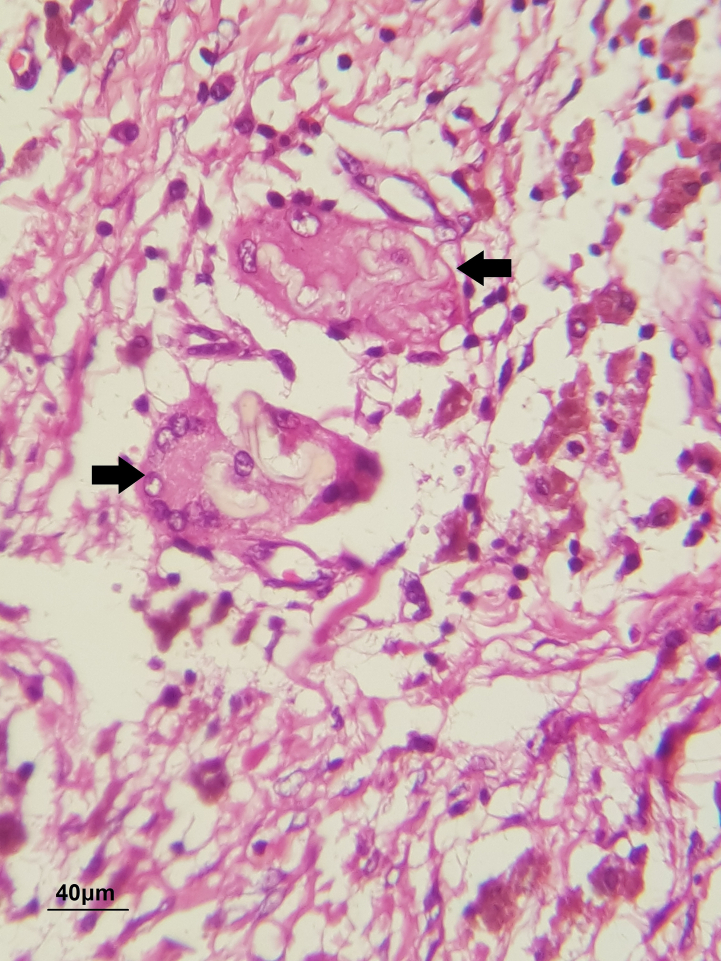
H&E staining reveals Giant cells swallowed distorted membranous materials (black arrow heads) (scale bar = 40 μm, 40× magnification).

**Fig. 2:**
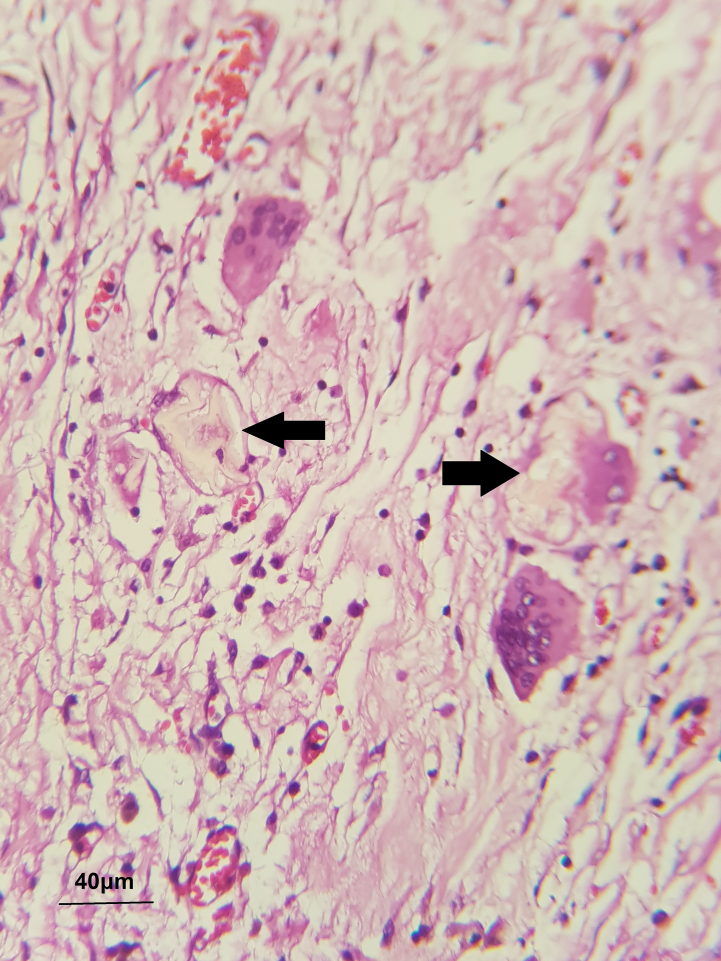
Swallowed enucleated fatty membranes (black arrow heads) (scale bar = 40 μm, 40× magnification).

**Figs. 3 and 4:**
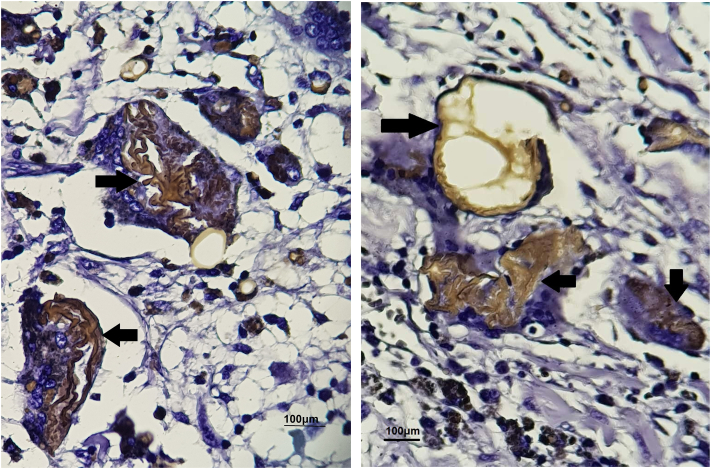
Brown color demonstrates necrosis (black arrows) in membranes in Sudan black staining (scale bar = 100 μm, 100× magnification). (For interpretation of the references to color in this figure legend, the reader is referred to the web version of this article.)

Regarding pathology, eosinophilic, crenulated fat membranes in macrophages and giant cells could be seen in H&E staining (Fig. 1, Fig. 2) Moreover, positive Sudan-black staining revealed lipids, memberanous material, and necrosis which are demonstrated by brown color (Figs. 3 and 4). SBB, LFB, long Ziehl-Neelsen, and D-PAS were also positive, confirming the diagnosis of MFN in gall bladder samples.

The article has been reported in line with the SCARE criteria [8,9].

## 3 Discussion

### 3.1 MFN etiology

Fatty cells, also known as adipocytes, are distributed in human organs. Fat necrosis, like every necrosis, is directly related to vascular circulation. Hypoxia leading to ischemia is a trigger for fat necrosis. After ischemia occurs, adipose cells are fragmented which creates intracellular vacuoles. The necrotic lipid in such vacuoles attracts accumulation of fibroblasts, multinucleated giant cells, and lipid-laden or foamy histiocytes (“fat-filled macrophages” or “foam cells”). Although phagocyted membranous is a characteristic feature of MFN, it was not reported in some cases. Moreover, the lipase released by enucleated adipocytes causes triglyceride breakdown and the presence of fatty acid in the interstitium. As a result, membranous fat necrosis happens when liquefactive necrosis causes cavity formation. The fibrinogen from damaged vessels is turned to active fibrin by thrombin leading to fibrosis. Furthermore, combination of negatively charged fatty acid and positively charged calcium ions develops calcification in necrotic fat tissue. However, the greater the ischemia, the greater the damage and necrosis will be. Hence, fibrosis and calcification are reported in cases of breast membranous fat necrosis [[10], [11], [12]].

### 3.2 MFN of abdominal lesions

Despite MFN rarity in intra-abdominal lesions, it is commonly reported in pancreatitis patients. Enzymic fat necrosis, also known as intra-abdominal fat necrosis, is the characteristic diagnosis of pancreatic disorders [[3], [4], [5], [6]]. Furthermore, there are some reports of microcytic MFN in appendix epiploica manifested by calcified fibrous nodules on the colon surface or in the abdominal cavity [11]. Nonetheless, MFN or lipo-membranous fat necrosis is commonly reported in subcutaneous and connective tissue such as dermal lesions, breast lumps, and osteodysplastic disorders [6,7,11].

Following the same etiology, circulatory disturbances and ischemia in cholecystitis is the most considered underlying cause in MFN presentation. The degraded fatty liquid is detected as foreign body. It is supported by the presence of macrophage and phagocytized products in necrotic fat tissue. Our patient suffered from diabetes mellitus and hypertension which could be the risk factors for MFN. Accumulation of inflammatory cells can shape MFN. Subsequently, such inflammatory response ends in fibrosis and hemorrhage in the affected organ, naming gall bladder [1]. Nevertheless, MFN in abdominal organs has no considerable clinical or imaging presentation and it is diagnosed by histological investigations [3,10,11].

### 3.3 MFN pathology

Membranous fat necrosis is a pathological term. In fact, diagnosis of MFN relies on histology. Membranocystose-like feature of MFN was first introduced in the osseous cystic lesions of a Japanese man in 1961. Macroscopically, hemorrhage, bright yellow liquified fat, necrotic area, calcification, and fibrosis can be seen [1,2,7]. Microscopically, in early stages of MFN due to phagocytosis, fatty tissue with hemorrhagic foci, enucleated adipocytes, foamy histocytes, and multinucleated giant cells can be detected [[12], [13], [14]]. Scattered MFN in fatty tissue could represent early stages of fat necrosis whereas fibrosis is representative of a chronic phase. In a case of MFN in gall bladder, several cystic sinuses, hemorrhages, thickened wall, fibrosis, and hemosiderin filled macrophages were detected. Fine membranous yellowish foreign bodies, liquefied fatty necrosis, were detected by D-PAS staining in cytomplasm of focally gathered giant cells [1]. MFN is defined by eosinophilic, crenulated membranes in H & E staining. Moreover, SSB staining can detect membranous materials in giant cells. Luxol fast blue (LFB) is also positive in MFN. Immunohistochemically, CD 68 is positive in the cytoplasm of giant cells in contrast to being negative in membranous substances. So, positive SSB, D-PAS, LFB, and *Z*-N are diagnostic for membranous fat necrosis [1,10,11,13,14].

## 4 Conclusion

Membranous fat necrosis is a pathological finding of fatty tissue, mostly in skin disorders and breast lesions. Till now, the real underlying cause of MFN is unknown. Thus far, this is the second case of membranous fat necrosis of gall bladder. Further studies are essential to investigate the pathophysiology of MFN in gall bladder.

## Ethical approval

Not applicable.

## Funding

This study received no funding.

## Author contribution

Authors' contributions: Dr. Mahsa Salehi and Sheidaei provided the main idea behind this research. Dr. Mahsa Salehi, Jafari, Rehman, Nourbakhsh, and Mehrnaz Salehi gathered data. Dr. Mahsa Salehi and Jafari wrote the manuscript and did the final revision and editions. Dr. Sheidaei submitted the manuscript.

## Guarantor

Dr. Mahsa Salehi.

## Registration of research studies

N/A

## Consent for publication

Written informed consent was obtained from the patient's guardian for publication of this case report and any accompanying images. A copy of the written consent is available for review by the Editor-in-Chief of this journal.

## Availability of supporting data

Not applicable.

## Authors' information

It is provided.

## Conflict of interest statement

There is no conflict of interest.
